# Secondary lesions of the mucous membrane of the oral cavity as a side effect of complex anticancer treatment: a literature review

**DOI:** 10.25122/jml-2023-0060

**Published:** 2023-11

**Authors:** Nazariy Ivanovych Helei, Vira Mykhaylivna Helei, Igor Valentynovych Zhulkevych

**Affiliations:** 1 Department of Surgical Dentistry and Clinical Disciplines, Uzhhorod National University, Ternopil, Ukraine; 2 Department of Oncology Radiology Diagnostics and Therapy and Radiation Medicine, I. Horbachevsky, Ternopil National Medical University, Ternopil, Ukraine

**Keywords:** mucositis, oral cavity, prevention, tumors, treatment

## Abstract

Today, both Ukraine and the world at large are faced with a significant number of oncological diseases with various localizations. The current state of diagnosis, prevention, early detection, and access to treatment leads to a substantial number of people in each country’s healthcare system who require comprehensive cancer treatments. Modern medical and diagnosis protocols in oncology involve the usage of ionizing radiation and aggressive toxic chemotherapeutic agents which can significantly disrupt the physiology of the mucous membrane of the digestive tract during treatment, especially of the oral cavity. The most common complication of complex anticancer therapy is the development of various lesions of the oral cavity, including mucositis, which harms the patient's quality of life, limits the doses of chemotherapy and radiation therapy the patient can receive, and also negatively affects the effectiveness of complex therapy treatment. Acute oral mucositis is observed among almost 100% of treatment cases. This is a significant problem for clinical oncology as it may also reduce patient compliance with comprehensive anticancer treatment. The results indicate the presence of oral problems in 100% of patients receiving specialized antitumor chemotherapy and radiotherapy, as well as the presence of a high need for specialized dental treatment.

## INTRODUCTION

According to official medical statistics conducted in several countries, there is a consistently high number of oncological diseases worldwide. Due to this fact, oral mucositis can generate several urgent healthcare problems. As stated by the World Health Organisation (WHO), mortality from oncological diseases ranks second after cardiovascular illnesses, therefore prevention, timely diagnosis, treatment, palliative care, and complex rehabilitation are extremely important factors for ensuring a high quality of life for patients. It is worth noting that the role of the dentist in patient rehabilitation is currently not properly assessed [1-3].

The incidence rates of oncological diseases are quite grim, as more than 10 million new cases are detected every year worldwide. In the mortality structure of the population of developed countries, malignant neoplasms occupy the third place and reach 13%. At the same time, the morbidity rate continues to rise. According to public health experts, the number of patients with malignant neoplasms can reach up to 1.4% of the country's population [4-6].

One of the most common complications of complex anticancer therapy is the development of oral mucositis, which negatively affects the patient's quality of life and limits the doses of chemotherapy and radiotherapy that the patient can receive in the future. Moreover, this can further negatively impact the effectiveness of complex therapy, and increase the cost of patient rehabilitation. In addition, mucositis of the oral cavity significantly rises the costs of treatment and the length of hospitalization for oncological patients. In this regard, medical science faces the task of finding and developing highly effective means of prevention and treatment for the various forms of oral mucositis to increase the effectiveness of antitumor treatment for oncological diseases. A clinical examination of the oral cavity of patients on anticancer therapy in an oncological hospital revealed poor oral hygiene and necessary specialized dental treatment. Among the complications that occur in the maxillofacial region as a result of radiation therapy, the most common are radiomucositis, radioepitheliitis, xerostomia, dysgeusia or parageusia, pain symptoms, erosions and ulcers on the mucous membrane, cheilitis, and purulent-necrotic lesions of the gums. A burning sensation of the oral mucosa was present in 92.3% of patients, paresthesia in 97.7%, and taste disturbance and xerostomia were present in all patients. In 30% of the cases there was angular cheilitis, 96.2% experienced bleeding gums, and 63.9% of patients presented the formation of ulcers of the oral mucosa. Out of all the patients, 13.1% needed dental care. In the year before hospitalization, patients had an average of 0.8±0.4 dentist visits per year, and only 9.2% of them used additional personal hygiene products for oral care [7-9]. The mucous membrane of the gastrointestinal tract, which passes into the mucous membrane of the oral cavity, is extremely vulnerable to stomatotoxicity, which is a consequence of radiation therapy. This tendency to localization arises as a result of many risk factors, such as a higher level of cellular metabolism, a different and heterogeneous microflora, the presence of chronic foci of infection of odontogenic origin, and trauma to soft tissues as a result of functional load. Complications can aggravate the patient's condition, while the untimely and inappropriate correction of complications can lead to the termination of anticancer treatment or the modification of the treatment scheme to ensure its effectiveness, which in the future can reduce the therapeutic effect and worsen the treatment prognosis for malignant neoplasms [10].

## MATERIAL AND METHODS

The research on the selected topic involved a thorough examination of various sources, including specialized literature, catalogs, and bibliographic references from the Scientific Library of the Uzhhorod National University of Higher Education and Science, as well as dissertations and abstracts. In addition, we also conducted a search in patent databases, such as those maintained by the State Institute of Intellectual Property of the Ministry of Justice of Ukraine and "Google Patents." Furthermore, we explored open data from active scientific councils specializing in dentistry, oncology, and radiography diagnostics and radiation therapy across Ukrainian and European Union institutions. Annual reports and bulletins from the Institute of Cancer of the Ministry of Health, Ukraine were also reviewed. In addition to these sources, directed searches were carried out on the internet and in scientific databases, including Medline, PubMed, Cochrane Database of Systematic Reviews, Scopus, Web of Science, SciElo, and Google Academic. The literature search was conducted using relevant keywords, including "malignant tumor," "treatment," "complication of treatment," "local and general consequences of treatment," "dental status," "chemotherapy," "radiotherapy," "mucositis," "radiomucositis," "oral microbiota," "regeneration of mucous membranes," "oral cavity rehabilitation," "radiation lesions," and "pharmacotherapy in dentistry."

## RESULTS

Following the search, 683 sources of information were analyzed for the period 2014–2018. Out of these, 139 sources corresponded to 90% of the suggested tags. The most published studies were in 2017 and 2018, with 112 and 146 research materials respectively. The evaluation outlined a constant interest in secondary lesions of the oral mucosa in patients with cancer.

## DISCUSSION

### Prevalence of mucositis

Despite the almost 100% prevalence of oral mucositis in patients with oncological diseases (especially with head and neck localization) who are treated according to various modern medical and diagnostic protocols, there are pronounced differences in the frequency and severity of their manifestations [11]. According to the European Society of Medical Oncology, the incidence of mucositis in grades III-IV cancers according to the WHO scale reaches 85% in patients after radiation therapy for tumors localized on the head and neck [11]. However, their clinic presentation differs in the context of the administration of various anticancer drugs. With all types of tumors, the use of chemotherapy with 5, 16 - fluorouracil, tegafur, or capecitabine has a high risk (20-30%) of leading to the development of mucositis of the digestive tract [12]. According to scientists, oral mucositis of III and IV-stage cancers can be observed in 70-80% of cases in patients with head and neck tumors receiving chemo-radiation therapy, and in 40-90% of cases in patients with oncohematological profile, after high doses of chemotherapy and bone marrow transplantation [12]. When using standard modes of fractionation of radiation therapy, mucositis occurs in more than 60% of cases. When using non-traditional modes of fractionation or a combination of radiation and chemotherapy, mucositis occurs in 100% of cases [12].

### Directions for solving the problem

Today, despite the wide availability of protocols for treating oncological patients, there is a lack of highly effective methods for preventing and treating the harmful side effects of complex chemotherapy on the oral mucous membrane.

Therefore, the innovation of complexes for the prevention and treatment of oral mucositis, which develops during antineoplastic therapy, is an urgent problem and a promising direction for oncology and dentistry. The prevention and treatment methods described in the literature are often complex and multi-component, requiring the use of expensive equipment and long-term training of specialists. Groups of drugs recommended in foreign treatment protocols are often imported and limitedly available in Ukraine [12-14].

### Pathogenesis of oral mucositis

Oral mucositis was defined as a purely local pathological process that developed as a result of the toxic effects of chemotherapy and/or radiation therapy on epithelial cells in a state of active proliferation [13]. It was believed that direct damage to epithelial cells leads to the loss of regeneration potential, atrophic changes, and the further development of ulcers. Modern authors consider this pathology as a more complex and cascading process [15]. S. Sonis *et al*. identified five development phases in the case of secondary lesions of the oral mucous membrane against the background of complex anticancer therapy [14]:

1. **Initiations**. At this stage, as a result of the direct effect of cytostatics or radiation therapy on epitheliocytes and cells of the submucosal layer, free radicals (activated oxygen atoms) are released into the tissues, which can damage the surrounding cells, tissues, and vascular network [15].
2. **Primary damage**. Free radicals at the cellular level cause irreversible damage to DNA chains, which leads to the death of some cells. Activation of the nuclear transcription factor (NFkB) in such a critical situation (response to DNA damage) promotes a cascade of protective reactions accompanied by the release of pro-inflammatory cytokines into the intercellular space, namely the tumor necrosis factor (TNFα), and interleukins IL1β and IL6. At the same time, other genes are activated, which increases the synthesis of molecules of adhesins, cyclooxygenase-2 (COX2), and stimulation of angiogenesis. In this phase of damage, the epithelial rate of regeneration decreases, which leads to atrophy of the mucous membrane and submucous layer [15].
3. **Formation and transmission of signals**. The activation of the transcription factor in the development of mucositis is not the only mechanism that initiates the apoptosis of epithelial cells. Simultaneously or sequentially, free radicals in the tissues activate the ceramide pathway of apoptosis - as a result of sphingomyelinase and ceramide synthetase activation, which directly cause programmed cell death. Also, fibronectin degradation acts as an activator of macrophages and matrix metalloproteinases (MMPs), which additionally activate TNFα and increase tissue damage. At this stage of the pathological process, significant pathological changes occur in the vital activity of the oral mucous membrane and the submucosal layer, although visually the tissues of the oral cavity may appear intact [16].
4. **Ulcer**. The most vivid phase in terms of clinical manifestations is represented by ulcers. Damage to the stem cells of the oral cavity epithelium leads to erosive changes in the mucous membrane. The ulcer acts as a focus of bacterial colonization, especially in the oral cavity, an area with a high level of bacterial contamination. Therefore, a secondary infection develops. Waste products and the remains of dead bacteria penetrate the submucosal layer and stimulate macrophages, which leads to the repeated release of pro-inflammatory substances into the tissues, namely cytokines. As a rule, this phase coincides with deep neutropenia in the patient, while individual bacteria can freely penetrate the submucosa, causing the development of bacteremia or even sepsis [15].
5. **Healing**. During this phase, the speed of epithelial regeneration (cell proliferation and differentiation), and normalization of the microbiome of the oral cavity and surrounding tissues occurs. This phase coincides with the normalization period of the patient's peripheral blood parameters and ends on the 15^th^-16^th^ day after the start of the course of chemotherapy (complex radio-chemotherapy) [16, 17].

When considering the features of radiation lesions of the oral mucous membrane (radiomucositis), early and late radiation damage is distinguished. Early radiation injuries include dry and wet necrosis of the epithelium of the oral cavity, and later atrophy of the epithelium, radiation fibrosis, and ulcers of the mucous membrane. Alteration processes prevail in the area of the radial ulcer of the oral mucosa. During the morphological examination, it can be found that the necrotic crust of the ulcer covers a sclerosed stroma, in which infrequent foci of round cell infiltration are present, leading to the expansion of small vessels. In contrast, larger vessels are visualized with thickened walls and an obliterated or narrowed lumen. The vascular factor comes to the fore in the radiation damage of the oral mucosa - changes in the permeability of blood vessels lead to hypoxia and disruption of the tropism of irradiated tissues with subsequent structural degradation of the mucous membrane. In the development of late radiation changes, damage to blood vessels with the subsequent violation of their permeability and microcirculation acquires leading importance. This leads to the release of blood plasma into the tissue and fibrinoid necrosis of vessel walls, obturation of their lumens, and the subsequent development of prolonged hypoxia. These changes are accompanied by a violation of tissue tropism, the development of dystrophic and destructive processes in tissues, which end in fibrosis or the formation of a radial ulcer in the affected areas. Also, the cause of radiation damage to vessels is not only necrosis of the endothelium, but also low proliferative activity of cells that retain viability, and a long period of their recovery (eight weeks). It is worth paying attention to the fact that with ionizing radiation of the oral mucosa, both blood and lymphatic vessels are affected. In the latter, obstruction with impaired lymph flow develops after radiation exposure [16-18].

### Comprehensive treatment of oncological diseases and secondary lesions of the mucous membrane of the oral cavity

In modern oncology, chemotherapy, along with surgical methods and radiation exposure, is one of the most important components of treatment protocols. The use of modern anticancer drugs in clinical practice is often accompanied by the development of adverse reactions. Among these, reactions caused by lesions of rapidly renewing tissues, in particular highly proliferative cells of hematopoietic and immunocompetent organs (bone marrow, gastrointestinal mucosa, hair follicles, etc.) predominate [19, 20]. Despite the development of the pharmaceutical industry, modern cytostatics remain non-selective and can damage almost all structures of the patient's body with varying frequency. Patients undergoing chemotherapy for malignant tumors experience certain complications related to the gastrointestinal tract - vomiting, nausea, diarrhea, constipation, enteritis, colitis, and mucositis with damage to the oral mucosa. Such complications can significantly aggravate the patient's condition. Untimely and inappropriate correction of the complications can lead to the termination of special treatment, and a change in the therapeutic scheme, which in the future can significantly worsen the therapeutic effect of chemotherapy, as well as change the prognosis of the disease in an unfavorable direction. In these patients, a decrease in the quality of life can be observed, necessitating additional examinations and treatment [21, 22].

According to the results of numerous clinical observations, mucositis, as a side effect of chemotherapy, can affect any part of the gastrointestinal tract. Damage to the oral cavity (acute stomatitis) usually occurs 4 to 16 days after the start of chemotherapy and can last 10-14 days after its completion. This can negatively impact the quality of life. Patients may experience long-term pain syndrome, which leads to a decrease in food intake with subsequent weight loss, affecting the duration of treatment. Mucositis can be one of the factors limiting the effective dose of chemotherapy and radiation therapy, which is manifested by inflammation and ulceration of the mucous membrane, as well as its submucous layer. The naturally pronounced proliferative activity of oral epithelial cells in the case of complex antitumor treatment makes them highly sensitive to the aggressive effect of cytostatic drugs. Also, in the development of secondary immunodepression, there is a high probability of experiencing a secondary infection, which leads to the development of not only local inflammatory lesions in the oral cavity but also more severe generalized infections. As evidenced by the literature, the frequency of lesions of the oral mucous membrane in patients with cancer is very high - up to 90%, and even with the use of standardized modern anticancer treatment protocols (polychemotherapy), it can vary within quite wide limits [23-25].

Other authors, in addition to acute mucositis (radioepitheliitis, radiomucositis) in focal and diffuse form, distinguish a separate lesion in the form of xerostomia in patients with cancer. It was separately observed that in individuals with inadequate oral hygiene and increased consumption of carbohydrates, as well as tobacco smokers and heavy alcohol consumers, chronic, inflammatory processes in the soft tissues of the oral cavity and bone tissue often occur or worsen [26].

One of the most encountered complications in patients with complex therapy of malignant tumors (in combination with radiation therapy) is radiation osteomyelitis of the jaws, the frequency of which can reach, according to various authors, up to 5–14% of cases. In its pathogenesis, several factors are decisive. Among them, the radiation level on tissues, the presence of mechanical damage to soft tissues and bones during or after radiation therapy, as well as the exacerbation of chronic odontogenic foci of infection, which can occur with the activation of pathogenic microflora, due to hygiene deterioration, are the most important ones. Therefore, the need for surgical rehabilitation of the oral cavity in a patient with a malignant tumor may arise both before and in remote periods after radiation therapy. According to several authors, the prevention of radiation osteomyelitis of the jaws also consists of special approaches to therapeutic, orthopedic, and surgical rehabilitation of the oral cavity [27].

### Modern approaches to the treatment of oral mucositis

To date, there are no universally accepted protocols for the prevention and treatment of oral mucositis. Correction of such a complication usually includes local and systemic treatment of the oral mucosa and the use of medicinal and non-pharmacological methods. Modern treatment of oral mucositis is mainly palliative, although the development of pathogenetic methods of correction is ongoing [26]. Analgesics represent the main symptomatic treatment since mucositis of the III-IV stage cancers is usually accompanied by severe pain, which significantly affects the patient's diet, oral care, and quality of life in general. That is why the prevention of mucositis pain is the leading link in the strategy to eliminate the inflammation of oral mucosa. According to WHO recommendations, depending on the intensity of pain, both systemic analgesics and local anesthetics are prescribed [27]. The research group "Mucositis" of the International Association for the Treatment of Cancer (MASCC) and the International Society for the Study of Oncological Diseases of the Oral Cavity (ISOO) developed clinical practical recommendations for the treatment of oral mucositis [28].

Treatment of oral mucositis is divided according to the following strategic directions:

- provision of adequate nutrition,
- control of pain sensations,
- use of antiseptics in the oral cavity,
- palliative treatment of xerostomia,
- prevention of bleeding in the oral cavity.

Today, several promising prevention and treatment strategies for oral mucositis and lesions of the oral mucous membrane have been identified: anti-inflammatory agents, antimicrobial drugs, biological response modifiers, antioxidants, and non-pharmacological interventions. Because the modern treatment of oral mucositis is partly pathogenetic and mainly symptomatic, the careful implementation of the doctor's recommendations regarding compliance with a high level of oral hygiene is defined as one of the most important preventive strategies. During the active course of chemotherapy and radiation, patients should avoid hot, spicy, solid food as well as alcohol, stop smoking, and minimize the use of removable dentures. The prevention of oral mucositis mainly boils down to urgent sanitation of the oral cavity before chemotherapy (in the presence of carious teeth and manifestations of inflammatory and inflammatory-dystrophic lesions of the periodontium). A rather original recommendation is the use of ice on a soft toothbrush immediately before and during the administration of cytostatics or radiation therapy, as well as a systematic examination of the mucous membrane of the oral cavity. However, the availability of data on the effectiveness of the listed recommendations in different clinics leaves the urgent problem of finding methods and means that allow minimizing the impact of an untreated oral cavity on the course of mucositis. Also, the issue of dynamic determination of the patient's dental status at the stages of radiation and chemotherapy and the identification of correlations between changes in the dental status and pathological changes in the oral mucosa is of great scientific interest [28, 29].

When considering specific recommendations during active treatment, it is advisable that the patient carefully moisturizes the oral cavity (rinsing, oral baths). The following agents are usually recommended for this purpose: 0.9% physiological sodium chloride solution, 25% sodium bicarbonate solution, weak saline solutions, 0.05% chlorhexidine solution, 3.0% hydrogen peroxide solution (diluted 1:1 with drinking water), and ready-made official solutions and sprays (Hexoral, Tantum-Verde). Some authors do not recommend the local use of chlorhexidine digluconate solutions for patients with oral mucositis, because they can inhibit mucosal regeneration. There is a fairly successful experience of using calcium phosphate in patients after high-dose polychemotherapy and transplantation of hematopoietic stem cells. However, this treatment method is limited due to the high cost. An alternative treatment route is the normalization of the microbiome of the oral cavity, which can reduce the risk of sepsis from resident or opportunistic microorganisms, an especially critical factor for patients with secondary immunodeficiencies after chemotherapy. Prophylactic doses of antibiotics applied locally are also recommended. In patients with cancer, solutions with polymyxin E, tobramycin, and amphotericin B were used during the entire course of radiation. Nonetheless, the use of antibacterial drugs (with cytotoxic properties) is undesirable in oncological and oncohematological patients, who already receive large doses of systemic immunosuppressive drugs. Therefore, the search for non-toxic treatment methods for the oral mucous membrane and the patient's body as a whole is a relevant direction for research [29, 30].

Separate recommendations for the treatment of mucositis also include the use of enveloping and keratoplasty agents, antioxidants - vitamin E, allopurinol solution, 2% methyl-uracil solution, sea buckthorn oil, propolis solution in milk, vitamin B12, chicken egg protein lysozyme, herbal decoctions (plantain), poultices based on an aqueous solution of furatsilin, and Maaloks [31]. One of the main directions in the treatment of oral mucositis caused by chemo-radiation is the use of anti-inflammatory drugs and prophylactic use of benzydamine compounds (Tantum Verde). Separate experimental studies point to the prospect of using anti-inflammatory cytokines, in particular Inteleykin-11 [31]. Another area of experimental research is the study of immunomodulators and drugs that accelerate the processes of proliferation and differentiation in the case of radiation. The direction of gene and replacement therapy today is an example of the use of recombinant human keratinocyte growth factor-1 (Palifermin) in medical practice [32].

## CONCLUSION

The population of Ukraine and the world at large faces a significant number of oncological diseases of various localization. The current level of diagnosis, prevention, and early detection (as well as access to treatment) leads to an insufficient number of people on complex anticancer treatment worldwide. Modern treatment and diagnostic protocols in oncology involve the use of ionizing radiation and aggressive toxic chemotherapeutic agents, which, during treatment, can noticeably disrupt the physiology of the mucous membrane of the digestive tract, and the oral cavity in particular. Acute mucositis of the oral cavity is observed in almost 100% of treated cases and is a significant problem for clinical oncology, as it can disrupt the patient's quality of life and reduce their compliance with complex anticancer treatment. At the current stage of the development of stomatology and oncology, there are no developed and verified protocols for the prevention and treatment of acute oral mucositis. The treatment is mostly symptomatic (partially pathogenetic), has low efficiency, and is quite expensive. The development of preventive measures (elimination of irritating factors, treatment of local chronic infection), the use of complex anti-inflammatory agents, and the correction of the cytokine profile of the course of the inflammatory process in the mucous membrane of the oral cavity are promising avenues to be explored.
